# Hidden viral proteins: How powerful are they?

**DOI:** 10.1371/journal.ppat.1011905

**Published:** 2024-01-18

**Authors:** Fangfang Li, Mingxuan Jia, Aiming Wang

**Affiliations:** 1 State Key Laboratory for Biology of Plant Diseases and Insect Pests, Institute of Plant Protection, Chinese Academy of Agricultural Sciences, Beijing, China; 2 London Research and Development Centre, Agriculture and Agri-Food Canada, London, Ontario, Canada; Mount Sinai School of Medicine, UNITED STATES

Viruses are submicroscopic obligate intracellular parasites and usually have tiny genomes. Based on the nature of their genomic nucleic acid and replicative intermediates, viruses may be classified into 7 classes, including positive-sense single-stranded RNA (+ssRNA), negative-sense single-stranded RNA (−ssRNA), double-stranded RNA (dsRNA), single-stranded DNA (ssDNA), and double-stranded DNA (dsDNA) viruses, retroviruses, and pararetroviruses. The vast majority of known viruses belong to the +ssRNA class with monopartite, bipartite, or tripartite ssRNA genomes (https://talk.ictvonline.org/taxonomy/). Infection by +ssRNA viruses can cause substantial damage to their host organisms, often leading to severe economic losses and, in some cases, even catastrophic mortality. Examples include human and animal infecting viruses such as acute respiratory syndrome coronavirus 2 (SARS-CoV-2), responsible for the ongoing COVID-19 pandemic, dengue, Zika, yellow fever, hepatitis C, foot-and-mouth disease, polio, chikungunya, SARS, and MERS viruses [1]. Many plant-infecting +ssRNA viruses, such as tomato brown rugose fruit virus, potato virus Y, plum pox virus, and maize dwarf mosaic virus, cause devastating diseases in diverse crops and threaten global food security.

The ssDNA viruses include some of the smallest and simplest viruses, with genomes only approximately 2 to 6 kb in length. Geminiviruses and nanoviruses are 2 known plant ssDNA viruses belonging to the families *Geminiviridae* and *Nanoviridae*, commonly known as geminiviruses and nanoviruses, are among the most economically important plant-infecting ssDNA viruses.

Due to the small genome size with limited coding capacity, viruses have to rely on host cellular pathways and hijack host factors for infection. On the other hand, viruses have evolved a variety of expression strategies to maximize the coding capacity of their compact genome. These noncanonical translation strategies adopted by viruses include internal ribosome entry, leaky scanning, non-AUG initiation, ribosome shunting, reinitiation, ribosomal frameshift, and stop-codon readthrough [2].

In recent years, “hidden” protein-coding open reading frames (ORFs) with neglected noncanonical translation strategies have been discovered in the genomes of various viruses [1–6]. For example, a short ORF encoding a small peptide named P3N-PIPO that is required for viral cell-to-cell movement was found to be embedded within the 5′ terminal region of viruses in the genus *Potyvirus* of the family *Potyviridae* [5].

## +ssRNA viruses encode hidden proteins in their −RNA

Gong and colleagues recently revealed that +ssRNA viruses encode additional functional proteins in their (−)-strand replication intermediates (−RNA) [6], which challenges the current consensus that −RNA lacks coding capacity. This surprising discovery raises an important question: To what extent is this expression mode shared among viruses?

**Fig 1:**
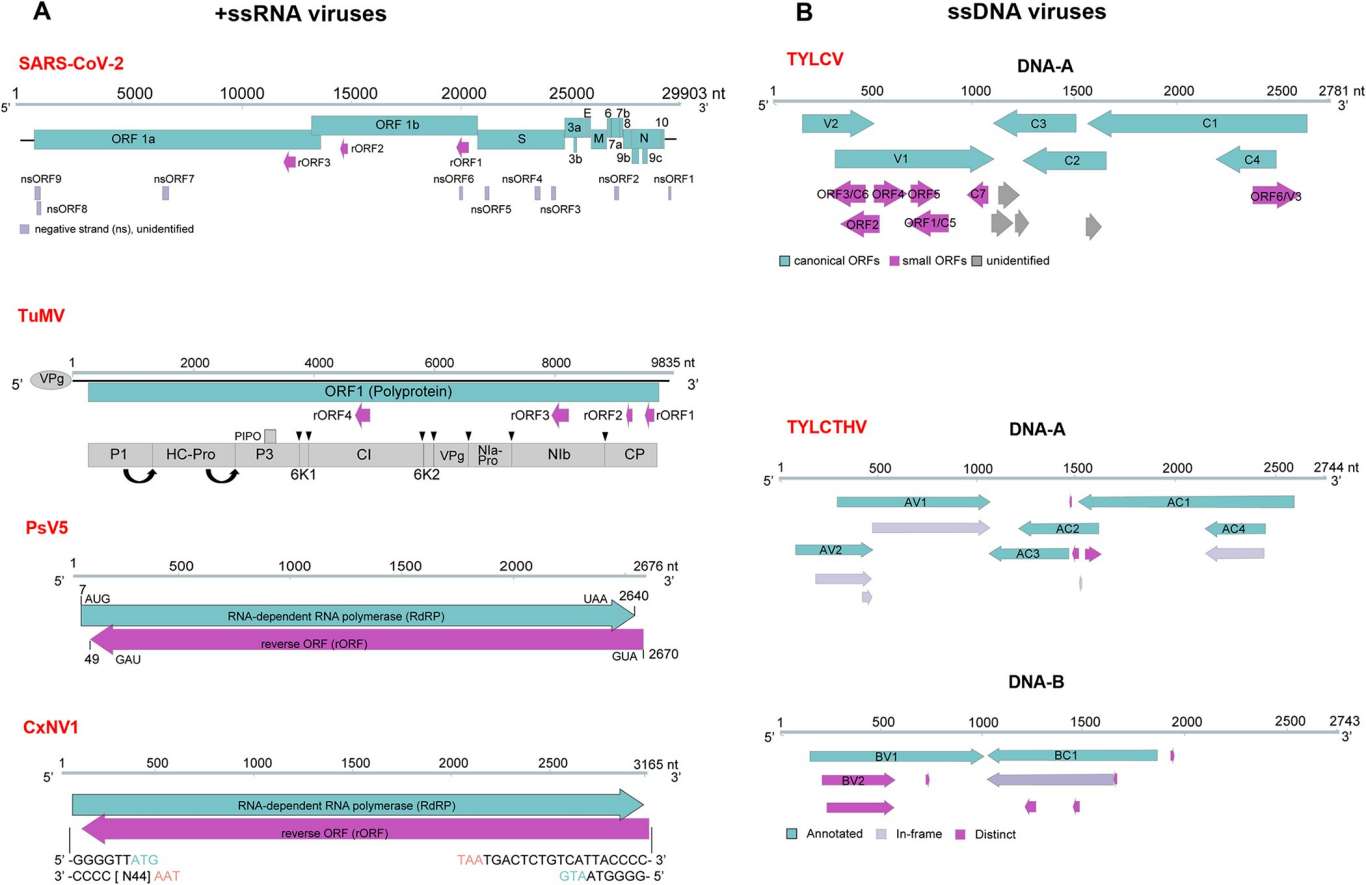
Viruses contain hidden open reading frames (ORFs) in their genomes. **(A)**Schematic diagram of the known ORFs and the predicted reverse ORFs (rORFs) in the viral positive-strand RNA (+RNA) and minus-strand RNA (−RNA) of positive-sense strand RNA (+ssRNA) viruses, respectively. (**B**) Schematic diagram of the known ORFs and the newly identified ORFs in the viral strand and the complementary strand of geminiviruses (ssDNA viruses). CxNV1, Culex narnavirus 1; PsV5, *Puccinia striiformis* virus 5; SARS-CoV-2, severe acute respiratory syndrome coronavirus 2; TuMV, turnip mosaic virus; TYLCV, tomato yellow leaf curl virus; TYLCTHV, tomato yellow leaf curl Thailand virus.

+ssRNA viruses require the synthesis of minus-strand RNA (−RNA) using their genomic RNA as a template. Based on the current concept, all viral proteins are translated via viral genomic RNA (and subgenomic RNA), whereas −RNA is considered a viral replication intermediate devoid of any coding capacity. However, Gong and colleagues recently reported that animal and plant +ssRNA viruses contain multiple conserved small reverse ORFs (rORFs) in their −RNA (Fig 1A) [6]. The identified rORFs are very short, encode small peptides (<10 kDa), and overlap part of known coding ORFs. This may explain why they were not discovered until recently when sensitive comparative genomic and proteomics methods became available. Gong and colleagues showed that the small rORFs in the −RNA of SARS-CoV-2 could suppress type-I interferon (IFN-I) production and facilitate vesicular stomatitis virus infection. Using turnip mosaic virus (TuMV, the family *Potyviridae*) as a model of plant +ssRNA viruses, the authors demonstrated that small proteins encoded by the rORFs display specific subcellular localizations. The TuMV rORF2 protein, one of the most conserved proteins encoded by rORFs in the *Potyviridae* family—which encompasses the highest number of known plant-infecting RNA virus species—forms punctate granules that localize in the perinuclear region and colocalizes with viral replication complexes. rORF2 can directly interact with the TuMV RNA-dependent RNA polymerase (RdRp). Deletion of rORF2 is lethal to the virus, and ectopic expression of rORF2 rescues the deletion mutant [7]. These results establish that the −RNA strand of 2 unrelated +ssRNA viruses infecting organisms from different kingdoms of life encodes proteins with biological functions essential for viral pathogenesis and infection.

Most eukaryotic mRNAs are translated in a cap-dependent fashion. As mentioned above, viruses have evolved multiple noncanonical mechanisms for efficient translation of viral proteins. Different viruses may employ different translation strategies to satisfy their specific needs [2]. Gong and colleagues found that SARS-CoV-2 and TuMV may utilize IRES to recruit ribosomes and facilitate the translation of these rORF-encoded small proteins [6].

Narnaviruses are +ssRNA viruses that infect diverse organisms, including fungi, plants, protists, arthropods, and nematodes. Recently, a large ORF with a yet unknown translation mechanism was identified in the −RNA of different narnaviruses (Fig 1A) [1,3,4]. In this report, Dinan and colleagues conducted a systematical analysis of narnaviral sequences available in the public domain and found that long rORFs are widespread in one clade of narnaviruses and frequently occupy >95% of the genome, since these ORFs comprise some of the longest-known overlapping genes, indicating their broad relevance to understanding overlapping gene evolution and the de novo origin of genes [3,4].

Consistently, Zhang and colleagues reported that a positionally conserved rORF within the −RNA of a novel narnavirus, *Puccinia striiformis* virus 5 (PsV5) (Fig 1A), from the devastating wheat stripe rust fungus *P*. *striiformis f*. sp. *tritici* (Pst). They found that overexpression of this rORF enhanced the wheat’s susceptibility to Pst infection and also boosted *Fusarium graminearum* virulence [4], thus providing evidence for the biological relevance of this rORF.

Taken together, recently emerging evidence [1,3–6] subverts the previous cognition that +ssRNA viruses encode all proteins solely on the +RNA, and the −RNA of +ssRNA viruses does not encode proteins. The proteome of +ssRNA viruses shall be revisited for expansion, and the biological functions of these rORF-encoded proteins need to be investigated.

### +ssDNA viruses encode hidden proteins in the genome

Apparently, hidden viral proteins are not unique to +RNA viruses. Geminiviruses are phytopathogenic DNA viruses with circular ssDNA genomes and contain more than 520 species that are mainly transmitted by insect vectors such as whiteflies and leafhoppers. It has been well established that geminiviruses encode 6 to 8 canonical viral proteins by applying a traditional 10-kDa arbitrary threshold; however, mounting evidence showed that they also encode small hidden ORFs with biological functions. By reconsidering the protein identification criteria, Gong and colleagues identified several ORFs in the geminiviral genome that encode small proteins, including V3, C5, and C7, and disclosed their specific cellular localizations and virulence functions (Fig 1B) [7–9]. For example, they found that the C5 protein of tomato yellow leaf curl virus (TYLCV, a geminivirus) anchors to plasmodesmata (PD) in virus infection following trafficking from the nucleus along microfilaments in *Nicotiana benthamiana*. Furthermore, they showed that C5-mediated PD localization is conserved in 2 other geminiviruses [8]. This study solves a long-sought-after functional connection between PD and geminivirus cell-to-cell movement. Thus, these data warrant an exploration of such small proteins in other geminiviruses. Almost concomitantly, Chiu and colleagues identified several hidden ORFs from another geminivirus, tomato yellow leaf curl Thailand virus (Fig 1B), through translation initiation landscape profiling [10]. They further showed that the translation of different protein isoforms was required for the viral pathogenesis in tomato plants. Therefore, these independent findings support that +ssDNA viruses, such as geminiviruses, encode additional small functional proteins.

## Concluding remarks

Recent identifications and functional assays of hidden viral proteins encoded by different +ssRNA viruses and plant ssDNA viruses greatly expand the known proteome and shed new light on protein translation strategies. Among them, the finding that the transient replicative −RNA of +ssRNA viruses contains rORF is extremely impressive. Since eukaryotic organisms encode several types of RdRps, it would be interesting to determine if any of them could transcribe cellular mRNAs and the resulting −RNAs also encode such hidden proteins. With the technology development of nanopore sequencing and ribosome profiling, more unknown small proteins from diverse viruses, including dsDNA, dsRNA, and −ssRNA viruses, would be identified. Understanding the roles of such hidden proteins in the infection cycle would help develop new strategies for preventing and controlling these devastating viruses in animals and plants.
